# First Report of the Association of the Psyllid Vector *Bactericera trigonica* (Hemiptera: Triozidae) with ‘*Candidatus* Liberibacter Solanacearum’ in Italy

**DOI:** 10.3390/insects15020117

**Published:** 2024-02-06

**Authors:** Giorgia Bertinelli, Lorenza Tizzani, Fabio Mosconi, Vincenza Ilardi, Sabrina Bertin

**Affiliations:** CREA Research Centre for Plant Protection and Certification, Via C.G. Bertero 22, 00156 Rome, Italy; giorgia.bertinelli@crea.gov.it (G.B.); lorenza.tizzani@crea.gov.it (L.T.); fabio.mosconi@crea.gov.it (F.M.); vincenza.ilardi@crea.gov.it (V.I.)

**Keywords:** Psylloidea, Apiaceae, phloem-limited bacteria, spy insects

## Abstract

**Simple Summary:**

Psyllids are insects that represent a potential threat to the cultivation of several crops, mainly as vectors of the ‘*Candidatus* Liberibacter’ bacteria species. Surveys on the presence and abundance of insect vectors of pathogens can provide important information on the presence of related pathogens in a certain area, even before the appearance of disease symptoms in plants. The occurrence of psyllid vectors was investigated on carrot crops in the “Altopiano del Fucino” area (Abruzzo region), where the highest Italian carrot production occurs. This survey revealed, for the first time in Italy, the presence of psyllid *Bactericera trigonica* adults associated with ‘*Candidatus* Liberibacter solanacearum’ (Lso). This finding provided important evidence of the risks for Lso outbreaks and prompted further research to assess the spread and the incidence of the bacterium in crop cultivations in Italy.

**Abstract:**

Psyllids, members of the family Triozidae, represent a potential threat to the cultivation of solanaceous and apiaceous crops worldwide, mainly as vectors of the phloem-restricted bacterium ‘*Candidatus* Liberibacter solanacearum’ (Lso). The Lso haplotypes C, D and E are known to affect apiaceous crops, such as carrot and celery, in several European countries. In Italy, data on the incidence and natural spread of both Lso and psyllids have not been reported so far. In this study, the presence of the vectors was investigated in a main Italian district for carrot production, the “Altopiano del Fucino” area (Central Italy). Both occasional and regular surveys were carried out on a total of five carrot fields and one potato field in 2021 and 2022. *Bactericera trigonica* (Hodkinson), which is known to efficiently transmit Lso to carrots, was found to be well-established in the area. High levels of population density were recorded in the summer period (more than 100 adult specimens per trap caught every two weeks) and then sharply decreased after the carrot harvest, confirming the strict association of this psyllid species with crop availability. In 2022, 27.5% of the total tested psyllid samples resulted in being positive for Lso haplotypes D and E, the latter being prevalent. This survey revealed, for the first time in Italy, the presence of *B. trigonica* adults associated with Lso in carrot crops. Although this study was limited to a few fields located in one area, it provided important evidence of the risks for Lso outbreaks and prompted further research to assess the spread and incidence of the disease in apiaceous cultivations in Italy.

## 1. Introduction

Psyllids (Hemiptera: Psylloidea) are phloem-feeding hemipterans, which are regarded as serious pests in agriculture and forestry worldwide. Aside from being responsible for direct damage, such as leaf curling and discoloration and plants’ stunted growth, psyllids are also efficient vectors of phytoplasmas and phloem-limited bacteria [1,2]. In this context, several psyllid species belonging to the families Triozidae and Aphalaridae are known to transmit *Candidatus* Liberibacter solanacearum (Lso) in a persistent and propagative manner [3,4]. *Candidatus* Liberibacter solanacearum is a Gram-negative bacterium infecting a variety of crops in the families *Apiaceae* and *Solanaceae*. The correlated disease symptoms are leaf curling along with yellow and purple discoloration, root and shoot growth retardation, and secondary root proliferation [3]. To date, fifteen different haplotypes of Lso have been identified worldwide, often in association with different host plants and geographical regions.

Lso A–E are currently the most relevant haplotypes, being responsible for important yield and economic losses. Haplotypes A and B infect solanaceous crops, mainly tomato and potato crops, in the western and central United States and New Zealand [5]. In addition to yield losses in the field, infection in potato crops results in typical post-harvest symptoms, such as the discoloration of tubers and vascular necrosis, particularly evidenced after frying (disease called “zebra chip”) [6]. Lso A and B and their known psyllid vector *Bactericera cockerelli* (Šulc) are EPPO A1-quarantine pests, and their presence in Europe has not been confirmed yet [4,7].

The haplotypes C, D and E are currently the most widespread in Europe, where Lso was first described in 2008 [8]. These haplotypes affect apiaceous crops, including carrot (*Daucus carota*), celery (*Apium graveolens*), parsnip (*Pastinaca sativa)*, parsley (*Petroselinum crispum)*, chervil (*Anthriscus cerefolium)* and fennel (*Foeniculum vulgare)* [9]. It is known that haplotype C is mainly transmitted by *Trioza apicalis* Förster in Central-Northern Europe, while haplotypes D and E are present in Southern Europe and in the Mediterranean basin and are efficiently transmitted by *Bactericera trigonica* Hodkinson [10]. Haplotype D was identified for the first time in a carrot crop in the Canary Islands (Spain) [11]. The first report of haplotype E relates to celery cultivated in Spain [12], followed by its identification in carrot and potato plants [13]. To date, the presence of Lso haplotypes D and E is continuously expanding despite the phytosanitary measures. Aside from the identification of haplotypes D and E in Spain [14,15], the two Lso haplotypes were also found in France [16], Morocco [17], Greece [18], Portugal [19], Israel [20], Tunisia [21] and Turkey [22]. Lso is also expanding in the Balkan peninsula, as reported by Trkulja et al. in Serbia [23].

Studies on the distribution of Lso evidenced the presence of the haplotypes C, D and E in seeds of the *Apiaceae* family since ancient times, indicating that it reached an economically significant level only when the environmental conditions were favorable for the vector population to increase to a sufficient size to cause epidemics [24]. Indeed, Lso spread is strongly associated with psyllid distribution and probing behavior [25]. In the Mediterranean region, *B. trigonica* is known to be an efficient vector of Lso D and E thanks to the high feeding activity on apiaceous crops, such as carrot and celery, and the relatively easy access to the phloem sap of these host plants [26,27]. Other psyllid species that are common in the Mediterranean region have been investigated for their potential role in Lso D and E transmission. Namely, epidemiological studies in Spain highlighted the presence of *Bactericera tremblayi* (Wagner) and *Bactericera nigricornis* (Foerster) in carrot fields. Although both species were found at very low population densities, some of the captured specimens tested positive for the bacterium [28,29], raising concerns about their role in spreading the disease. Further studies showed that adults of *B. tremblayi* can settle on and feed from the phloem of carrots in the absence of a plant suitable for reproduction, such as leek, but then they failed to transmit Lso to other carrot plants [30]. In recent laboratory experiments, *B. nigricornis* was able to acquire and transmit haplotype E from carrot to carrot with a very high transmission rate [2]. Its vector role could be mitigated in the field by its clear host preference for potato compared to carrot; nevertheless, further research is needed to clarify how *B. nigricornis* contributes to Lso spread. 

Carrots are widely cultivated in Europe for both food consumption and seed production, and in 2021, the crop covered 229,347 ha and produced 8,860,389 t [31]. Italy remarkably contributes to the European carrot yield, being the fifth producer with a total area harvested of 10,680 ha and a production of 498,270 t. Carrot cultivation is guaranteed throughout the year by the summer and winter cultivation cycles from Northern to Southern Italy. Despite the importance of this crop, little information about the presence of both Lso and the vectors in Italy is currently available. In 2016, the presence of the bacterium was confirmed in carrot seeds [32], and a year later, in a few carrot plants in Sicily [33], but no further surveys have been carried out so far. Notices of the presence of *B. trigonica*, *B. nigricornis* and *B. tremblayi* refer to outdated records or museum collections only [34,35]. This lack of information can be a potential gap in the disease prevention and control of carrot crops. For this reason, a survey was carried out to investigate the psyllid populations and to assess the presence of Lso in the collected potential vectors. Such a survey was performed in the “Altopiano del Fucino” area (Abruzzo Region, Central Italy), where the highest Italian carrot production occurs (about 2300 ha/year for 60–70 t/ha; Regional Phytosanitary Service, personal communication, 2023), which also includes local varieties listed in the Protected Geographical Indication (PGI) specialties [36,37]. 

## 2. Materials and Methods

### 2.1. Sampling

**Figure 1:**
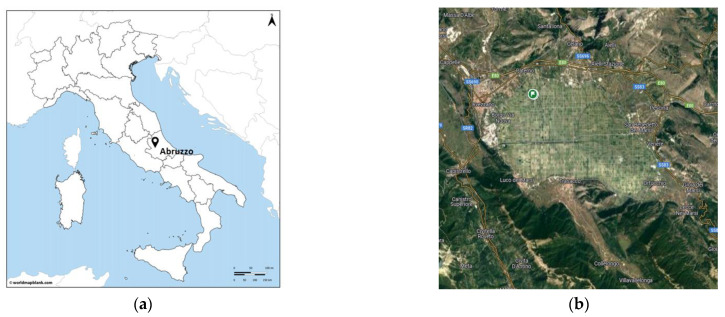

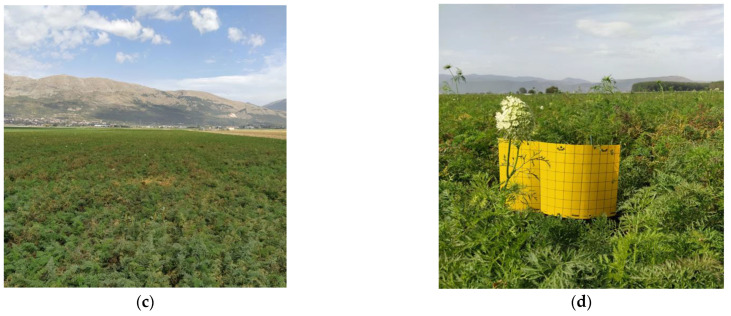
(**a**) Localization of the “Altopiano del Fucino” area (Abruzzo region) in Italy. (**b**) Details from a satellite over “Altopiano del Fucino” (Google Maps, Piana del Fucino, retrieved on 01/12/23). (**c**,**d**) Pictures of carrot field 1.

Surveys for psyllid vectors were carried out in 2021 and 2022 in a total of five carrot fields located in the “Altopiano del Fucino” area (Abruzzo region, Central Italy; Figure 1; Table 1). In the summer of 2021, a single sampling of adult psyllids was performed by sweep net at three different fields; two of the three fields were also surveyed using yellow sticky traps from 23/06/2021 to 12/07/2021 (Table 1). In 2022, two carrot fields and an adjacent potato field were regularly surveyed by yellow sticky traps from crop emergence (June) to harvest (early September) or early November 2022 to monitor the psyllid population, even in the absence of the host crop (Table 1).

Each sampling by sweep net consisted of ten consecutive sweeps at ten different points. Collected samples were frozen at −20 °C until morphological identification and then stored in absolute ethanol at 4 °C. 

Four to six sticky traps (25 cm high and 40 cm wide, divided into 120 squares of 8 cm^2^) were placed at the edges of each field at canopy level. The traps were replaced every fifteen days and shortly stored at 4° C until examination under a stereomicroscope (Wild Heerbrugg, Gais, Switzerland). The number of captured psyllids was estimated by counting the specimens on 60 squares evenly distributed on each side of the trap (120 squares/trap). Fifteen randomly chosen specimens per trap (at the most) were removed using organic solvent and stored in absolute ethanol at 4 °C as pools of five individuals each.

### 2.2. Psyllid Identification

Adult psyllids were identified at the genus/species level based on the morphological characteristics following the taxonomic keys of Hodkinson and White (1979) [38]; Hodkinson (1981) [39]; Burckhardt and Freuler (2000) [40] and Carnegie et al. (2017) [41]. The species identification was then confirmed by molecular analysis. 

The DNA for both psyllid species identification and Lso detection was extracted from both single and pooled specimens. In detail, the analyses were performed on 38 single psyllid specimens collected in 2021, 16 by sweeping net and 22 from yellow sticky traps. In 2022, the analyses were performed on one or two pools of five individuals collected from the same trap for a total of 98 psyllid pools: 62 from field 1, 22 from field 2 and 14 from field 3.

DNA extraction was performed using the GRiSP Xpert directXtract Lysis Buffer (Grisp research, Portugal) following the manufacturer’s instructions.

The molecular psyllid species identification was based on the ribosomal ITS2 sequence. End-point PCR was performed using the primers CAS5p8sFcm (5′-CGAACATCGACAAGTCGAACGCACA-3′) and CAS28sB1d (5′-TTGTTTTCCTCCGCTTATTAATATGCTTAA-3′) [42] in a final volume of 25 µL using PCR Master Mix (Promega) with a primer concentration of 0.5 µM. The cycling conditions were: one cycle at 95 °C for 5 min, 35 cycles at 95 °C for 30 s, 52 °C for 1 min and 72 °C for 1 min, followed by a final extension at 72 °C for 10 min. Amplicons were subjected to Sanger sequencing (BioFab research, Rome, Italy). Sequence identification was performed using the BLAST^®^ tool on the National Center for Biotechnology Information (NCBI) gene bank (https://blast.ncbi.nlm.nih.gov/Blast.cgi, accessed on 20/12/2023). 

### 2.3. Lso Detection and Haplotype Identification

Lso detection was performed following EPPO PM 7/143 (1) [9,43]. The first screening was performed by real-time PCR according to Li et al., 2009 (as reported in EPPO PM 7/143, Appendix 5) [9,44]. Samples were considered positive if an exponential curve was produced with a cycle threshold (Ct) value of < 40. For each amplification event, the following controls were included: a negative extraction control, a positive amplification control and a negative amplification control. Samples were tested in three technical repetitions. The positive samples were further analyzed for the Lso haplotype identification. End-point PCRs, according to Ravindran et al., 2011 (EPPO PM 7/143, Appendix 7 [9,45]) targeting the 16–23S rRNA intergenic spacer, and according to Munyaneza et al., 2009 (EPPO PM 7/143-Appendix 8 [9,46]), targeting the rplL-rplJ gene region (50S rRNA), were performed. The two amplified regions were sequenced and compared to reference sequences as indicated by EPPO PM 7/143 (1) [9]. Sequence alignment was performed using Clustal Omega [47] and the online BLAST^®^ tool (https://blast.ncbi.nlm.nih.gov/Blast.cgi, accessed on 20/12/2023).

## 3. Results

### 3.1. Occasional Survey of Psyllids in 2021 

The occasional surveys carried out in 2021 in the “Altopiano del Fucino” area by sweep net allowed a collection of five psyllid specimens in carrot field 1, one specimen in field 2 and ten specimens in field 3. The yellow sticky traps placed in the first two fields captured high numbers of psyllids, on average 500 psyllids per trap from field 1 and 100 psyllids per trap from field 2 (Table 1). All the collected specimens were identified as *B. trigonica* by means of morphology. The ITS2-sequence analysis, carried out on the single specimens caught by a sweeping net and on two single specimens from each trap (for a total of 10 individuals from field 1 and 12 individuals from field 2), confirmed the identity as *B. trigonica*. The GenBank accession number of ITS2 sequences from representative individuals of each sampling is reported in Table 1. 

### 3.2. Regular Survey of Psyllids in 2022

*Bactericera trigonica* was again the only psyllid species found in the two carrot fields and one potato field that were regularly inspected in 2022. For each field and date of survey, ITS2-sequence analysis was performed on representative specimens of each trap, and the obtained sequences confirmed the identity as *B. trigonica* (the GenBank accession numbers are reported in Table 1).

**Figure 2:**
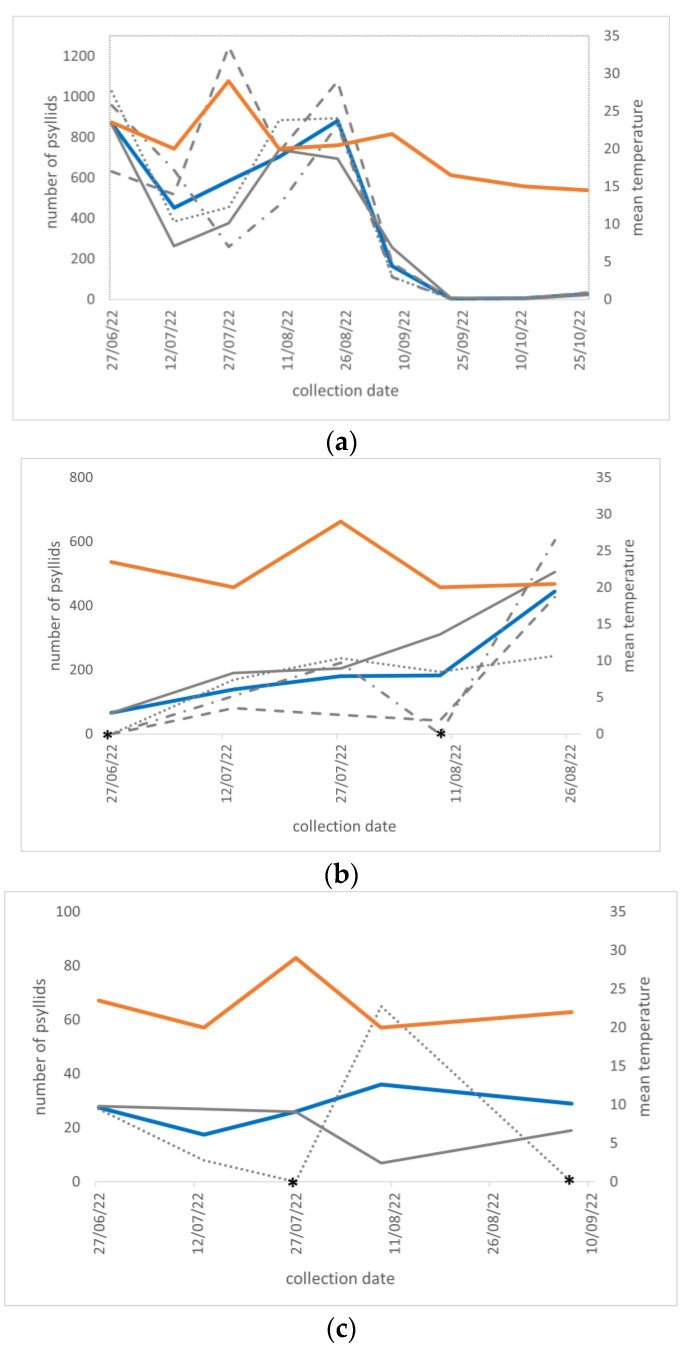
Population dynamics of *Bactericera trigonica* adults captured in 2022. (**a**) Field 1—carrot cultivation; (**b**) Field 2—carrot cultivation; (**c**) Field 3—potato cultivation. Gray lines: number of specimens counted at each time point per trap (four traps in fields 1 and 2, i.e., solid line = Trap 1, dotted line = Trap 2, dot-dash line = Trap 3 and dashed line = Trap 4; two traps in field 3, i.e., solid line = Trap 1 and dotted line = Trap 2); blue line: mean number of specimens across the traps; and orange line: mean temperature values. Asterisks: no data were collected for the time point.

The highest density of the *B. trigonica* population was recorded in carrot field 1, being a total of 14,772 specimens counted throughout the survey period, with at least more than 100 specimens caught per trap during the carrot growing season (Table S1). Two peaks of density were observed in late June (869.50 specimens per trap on average) and late August (an average of 881.75 specimens per trap), followed by a sharp decrease in the population in the middle of September after the carrot harvest (5.00 specimens per trap) (Figure 2a). Even if the adults counted after that period were a few units per trap, the psyllid continued to be present until early November. 

At the second site surveyed in 2022, a total of 3,676 adults of *B. trigonica* were collected in the carrot field until early September, when the harvest occurred (Table S2). The mean number of specimens per trap gradually increased from the minimum registered in June (66 specimens) to July and early August and reached the maximum peak (445.25 specimens) in late August (Figure 2b). At the same site, only 217 psyllid specimens were collected in the adjacent potato field (Table S3). The population accounted for a few tens of specimens, and this low population density was maintained throughout the season (Figure 2c).

### 3.3. Lso Detection and Haplotype Identification

All 38 single specimens analyzed in real-time PCR in 2021 (16 specimens collected by sweeping net and 22 from yellow sticky traps) tested negative for Lso. In 2022, a total of 27 out of 98 (27.5%) tested pools of five specimens were found positive for Lso in the real-time PCR. 

**Figure 3:**
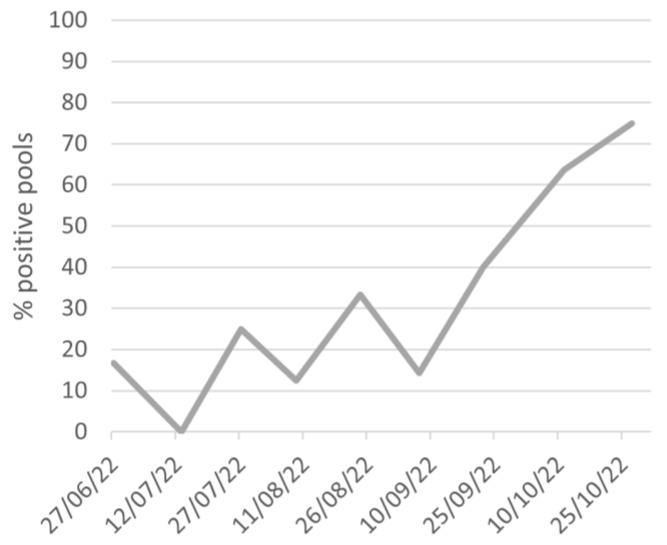
Temporal distribution of the percentage of psyllid pools that tested positive for Lso in field 1.

Most of the positive pools were from carrot field 1 (21 out of 62 tested pools), but some of them were also found in carrot field 2 (3 out of 22 tested pools) and the potato field (3 out of 14 tested pools). The percentage of Lso-positive samples found in field 1 progressively increased during the carrot growing season. The Lso-positive pools were less than 40% until the beginning of September and then rose up to 60–75% in October (Figure 3). The temporal distribution of the Lso infection rates could not be analyzed for fields 2 and 3 because of the low numbers of positive pools.

A total of 21 out of the 27 positive pools occurred in PCR amplification and sequencing for determining the Lso haplotype (Table 2). As foreseen by the EPPO standard PM7/143 (1) [9], it was not always possible to obtain sequences with both PCR methods for each pool tested; however, it was possible to identify the Lso haplotype at least with one PCR method. Among the sequenced PCR products obtained from the 21 positive pools, 17 corresponded to haplotype E and 4 to haplotype D (Table 2). The pools belonging to haplotype D were collected from field 1 only, whereas haplotype E was found in 14 psyllid pools from carrot field 1, two from carrot field 2, and two from the potato field. All 18 pools amplified by the Ravindran et al. 2011 test showed single-nucleotide polymorphisms (SNPs) with respect to the reference gene EU812559 (EPPO PM 7/143 (1)) [9]. In particular, two SNPs (G vs. A) were at the 1632 and 1648 positions of the reference gene for haplotype D, and one SNP (G vs. A) was at the 1620 position for haplotype E.

## 4. Discussion

Surveys on the presence and abundance of insect vectors have repeatedly proved to be incisive for revealing the introduction of pathogens in a new area or for monitoring the progression of pathogen spread [48,49]. Indeed, insects can be indicators (“spy insects”) of a pathogen’s presence in a certain area even before the appearance of disease symptoms in plants, playing an important role in disease surveillance. In the case of Lso, the disease is known to be widespread in several countries of the Mediterranean basin, where the presence of both bacterium and psyllid vectors was reported in cultivations of carrots and other apiaceous crops. Italy is an exception, however, since data have not been collected on the potential incidence and natural spread of the disease, although the interception of commercialized infected carrot seeds suggested the presence of haplotypes D and E [32]. Thus, field surveys were demanded, especially for the presence of the psyllid vectors that are solely responsible for an efficient plant-to-plant transmission after Lso introduction in a new area [30,50]. 

The occasional surveys carried out in 2021 in the “Altopiano del Fucino” area reported the presence of *B. trigonica*, one of the psyllid species that is recognized to be a fully competent vector of Lso on apiaceous crops [30,51]. The number of individuals captured by yellow sticky traps in a very short period suggested that populations of *B. trigonica* are well-established in this area where carrot and potato crops are intensively cultivated. The surveys also confirmed that the yellow sticky traps are very effective in capturing high numbers of psyllids and are more useful than the sweep net for detecting adult population peaks [52]. Therefore, the traps were chosen for the regular surveys in 2022, also considering that the state of conservation of the stuck specimens allowed their identification by means of morphology and/or molecular analysis.

High levels of population density were confirmed by the regular surveys carried out in 2022: more than 100 adult specimens per trap were caught every two weeks throughout the carrot growing season in both fields 1 and 2. These density levels were similar to those recorded in Spain, both in the mainland and the Canary Islands, where hundreds of psyllids were captured by different types of traps in carrot fields during the summer of several years [28,29]. In our survey, the population was particularly abundant in field 1, where two maximum peaks of around 800 individuals per trap were recorded in June and late August. The population of field 2 reached one peak in late August, just before the carrot harvest. Multiple density peaks were also observed in Spain at different timings and were correlated with both optimal mean temperatures and reduced insecticide treatments [29]. In the “Altopiano del Fucino” area, the mean daily temperatures, which are known to be optimal for psyllid development (around 25 °C), were actually registered in June and then in late August 2022, whereas the temperatures were much higher in July and early August and were thus less favorable for a high population density [53]. No insecticide treatments specifically targeting psyllids were performed in the considered area, but the routine chemical control of other insect pests, mainly aphids and the carrot fly *Chamaepsila rosa* [54], could have affected the trend of the population density of *B. trigonica*. Thus, it is not possible to exclude that the absence of the peak in June 2022 in field 2 could be due to the treatments performed against other targets in or near the surveyed site. 

After the maximum peaks in August, the populations of *B trigonica* sharply decreased as soon as the carrots were harvested in both fields 1 and 2. This supports the strict association between this species and apiaceous crops, as already observed in other surveys carried out in the Mediterranean basin [28,29,52,55]. Similarly, the low number of adults collected in field 3 further confirms that *B. trigonica* occurs at high levels of population density in the presence of apiaceous hosts only [28,29]. 

In field 1, the survey was prolonged until the end of October 2022, one month and a half after the carrot harvest. It was observed that the number of *B. trigonica* specimens decreased in the absence of the host crop but was never zero, and few specimens continued to be caught until the end of the survey despite the upcoming cold season. In the “Altopiano del Fucino” area, the mean daily temperature registered in October 2022 was 9.1 °C only, and the minimum monthly temperatures even went down to 0 °C [53]. This suggests that *B. trigonica*, which is known to prefer mild climates, can survive the low temperatures occurring in autumn in the “Altopiano del Fucino” area. Its extended presence in the field can also be favored by the availability of other apiaceous crops that are cultivated until late autumn, such as fennel. The behavior of this psyllid species during the next winter months and its settlement on plant species that differ from those commonly reported as hosts, still remains an open question in the Mediterranean basin [29]. Indeed, the migration aptitude of *B. trigonica* has not been deeply studied so far, and it is uncertain if adults overwinter on conifers or evergreen shrubs, as suggested for Central Europe [40]. 

Regarding the Lso presence in vectors, the occasional survey carried out in 2021 did not reveal the bacterium in the 16 specimens analyzed, while in 2022, when the regular surveys were performed, 27.5% of the total tested psyllid pools collected in the three fields resulted in being positive for Lso. This Lso incidence in *B. trigonica* is comparable with the percentages that occurred in Spain on celery and carrot crops [28]. 

The molecular characterization of Lso confirmed the association of *B. trigonica* with the haplotypes D and E, the latter being prevalent. *Bactericera trigonica* has been repeatedly demonstrated as a highly efficient vector of both haplotypes D and E in *Apiaceae* [26,30], especially the females that reached the phloem more times and tended to salivate longer than males [51]. It is known that this transmission efficiency can result in a rapid spread of the disease in a field. Indeed, an initial Lso prevalence of 3–4% has been estimated to increase up to approximately 90% after six months of carrot cultivation under a high population of infectious *B. trigonica* [27]. The temporal dynamic observed for the rate of Lso-positive psyllid samples in field 1 confirms this trend. Indeed, the highest rates of infectious psyllids were recorded in the late cycle of carrot cultivation and even after the carrot harvest, suggesting that Lso incidence in the crop was progressively increasing during the growing season. Thus, the transmission efficiency of *B. trigonica* combined with the observed high psyllid population density suggests that the incidence of Lso in carrot fields 1 and 2 could be potentially high [26,30]. 

*Bactericera trigonica* was the only psyllid species collected in the surveyed fields in 2021 and 2022. In other Mediterranean countries, such as Spain and Tunisia, this species was frequently found in mixed populations with *B. tremblay* and *B. nigricornis,* which are also noticed for their competence in acquiring and/or transmitting Lso [28,29,30]. Notably, in Spain, *B. nigricornis* has been found in carrot crops and, most abundantly, in potato crops [52]. Aside from its preference to settle on *S. tuberosum* plants, this species also proved to efficiently transmit Lso from carrot to potato plants under controlled conditions [2]. For these reasons, *B. nigricornis* is regarded as a potential threat to potato cultivation, raising concerns for Lso outbreaks within this crop. During the present survey, no specimens of *B. nigricornis* were captured, not only in carrot fields but also in potato field 3. The absence of *B. nigricornis,* as well as the previous experimental evidence of low efficiency of Lso transmission from carrot to potato by *B. trigonica* [30], suggest a limited risk of Lso spreading in potato cultivations within the “Altopiano del Fucino” area.

## 5. Conclusions

The survey carried out in the “Altopiano del Fucino” area revealed for the first time in Italy the presence of *B. trigonica* adults associated with Lso on carrot crops. Although the study was limited to a few fields located in one area, it provides important evidence of the risks for Lso outbreaks and prompts further research to assess the spread and incidence of the disease in apiaceous cultivations in Italy. First, the level of plant infection in carrot and other apiaceous crops is under investigation. Second, further surveys are ongoing to increase our knowledge of psyllid phenology and demography in both apiaceous and solanaceous crops in several Italian areas.

## Figures and Tables

**Table 1:** Occasional and regular surveys of adult psyllids associated with carrot and potato cultivations in the “Altopiano del Fucino” area (Abruzzo region; Central Italy) in 2021 and 2022.

Year of Sampling	Sampling Site (Geographical Coordinates)	Host Plant	Sampling Method (Number of Traps per Field)	Period of Sampling	Total Number of Captured Adult Psyllids	GenBank Accession Number for Psyllid ITS2 Sequence
2021 (occasional survey)	Field 1 (42°2′20.4′′ N 13°31′30′′ E)	Carrot	Sweep net	23/06/2021	5	OR978342
			Yellow sticky traps (5)	23/06/2021–12/07/2021	250	OR978345
	Field 2 (42°0′7.2′′ N 13°32′27.6′′ E)	Carrot	Sweep net	23/06/2021	1	OR978343
			Yellow sticky traps (6)	23/06/2021–12/07/2021	380	OR978346
	Field 3 (41°58′48′′ N 13°33′21.6′′ E)	Carrot	Sweep net	12/07/2021	10	OR978344
2022 (regular survey)	Field 1 (42°2′16.8′′ N 13°29′24′′ E)	Carrot	Yellow sticky traps (4)	13/06/2022–27/10/2022	14,772	OR978347; OR978348; OR978352; OR978355; OR978356; OR978357; OR978358; OR978359
	Field 2 (42°0′00.6′′ N 13°36′25.2′′ E)	Carrot	Yellow sticky traps (4)	13/06/2022–07/09/2022	3676	OR978349; OR978350; OR978353; OR978354
	Field 3 (42°0′3.6′′ N 13°36′25.2′′ E)	Potato	Yellow sticky traps (2)	13/06/2022–09/08/2022	217	OR978351

**Table 2:** Lso haplotype identification for the pools of five *B. trigonica* specimens that tested positive in real-time PCR according to [44], and that were amplified by PCR according to [45] and/or [46]. Related GenBank accession numbers are reported; N.D.: not determined.

Field and Host Plant	Pool N	Lso Haplotype by Ravindran et al., 2011 [45] (GenBank Accession Number)	Lso Haplotype by Munyaneza et al., 2009 [46] (GenBank Accession Number)
Field 3, potato	21	E (OR962166)	E (OR983295)
Field 1, carrot	23	E (OR962167)	N.D.
Field 1, carrot	69	E (OR962168)	N.D.
Field 2, carrot	102	E (OR962169)	N.D.
Field 2, carrot	106	E (OR962170)	E (OR983296)
Field 3, potato	112	E (OR962171)	N.D.
Field 1, carrot	122	E (OR962172)	N.D.
Field 1, carrot	147	E (OR962173)	N.D.
Field 1, carrot	158	D (OR962162)	D (OR983292)
Field 1, carrot	160	D (OR962163)	D (OR983293)
Field 1, carrot	162	N.D.	E (OR983297)
Field 1, carrot	166	D (OR962164)	N.D.
Field 1, carrot	168	N.D.	E (OR983298)
Field 1, carrot	169	E (OR962174)	N.D.
Field 1, carrot	177	N.D.	E (OR983299)
Field 1, carrot	188	E (OR962175)	E (OR983300)
Field 1, carrot	191	E (OR962176)	N.D.
Field 1, carrot	192	D (OR962165)	D (OR983294)
Field 1, carrot	194	E (OR962177)	N.D.
Field 1, carrot	197	E (OR962178)	E (OR983301)
Field 1, carrot	198	E (OR962179)	E (OR983302)

## Data Availability

Publicly available datasets were analyzed in this study. This data can be found here: https://www.ncbi.nlm.nih.gov/nucleotide/, accessed on 05/02/2024. Accession numbers are reported in Table 1 and Table 2.

## Supplementary Materials

The following supporting information can be downloaded at: https://www.mdpi.com/article/10.3390/insects15020117/s1, Table S1: Number of psyllids collected per trap at each time point infield 1 in 2022. Table S2: Number of psyllids collected per trap at each time point in field 2 in 2022, Table S3: Number of psyllids collected per trap at each time point in field 3 in 2022.
